# MRI assessment of cortical thickness and functional activity changes in adolescent girls following three months of practice on a visual-spatial task

**DOI:** 10.1186/1756-0500-2-174

**Published:** 2009-09-01

**Authors:** Richard J Haier, Sherif Karama, Leonard Leyba, Rex E Jung

**Affiliations:** 1 School of Medicine (Emeritus), University of California, Irvine CA, USA; 2 McConnell Brain Imaging Center, Montreal Neurological Institute, McGill University, Montreal, Canada; 3 Mind Research Network, Albuquerque, New Mexico, USA; 4 Department of Neurosurgery, University of New Mexico, Albuquerque, New Mexico, USA

## Abstract

**Background:**

Neuro-imaging studies demonstrate plasticity of cortical gray matter before and after practice for some motor and cognitive tasks in adults. Other imaging studies show functional changes after practice, but there is not yet direct evidence of how structural and functional changes may be related. A fundamental question is whether they occur at the same cortical sites, adjacent sites, or sites in other parts of a network.

**Findings:**

Using a 3 T MRI, we obtained structural and functional images in adolescent girls before and after practice on a visual-spatial problem-solving computer game, Tetris. After three months of practice, compared to the structural scans of controls, the group with Tetris practice showed thicker cortex, primarily in two areas: left BAs 6 and 22/38. Based on fMRI BOLD signals, the Tetris group showed cortical activations throughout the brain while playing Tetris, but significant BOLD decreases, mostly in frontal areas, were observed after practice. None of these BOLD decreases, however, overlapped with the cortical thickness changes.

**Conclusion:**

Regional cortical thickness changes were observed after three months of Tetris practice. Over the same period, brain activity decreases were observed in several other areas. These data indicate that structural change in one brain area does not necessarily result in functional change in the same location, at least on the levels assessed with these MRI methods.

## Findings

Plasticity of gray matter (GM) in adults has been demonstrated before and after practice of both motor and cognitive tasks [1-3]. Evidence points to plasticity effects for both novel learning of a task and for continued practice of a task once learned [4]. A fundamental question is whether GM increases after practice in a specific brain area are concomitant with functional changes in the same area (site specific), and/or whether different areas in the same network show changes. A secondary question is whether GM increases are associated with increased or decreased brain activity. Functional imaging studies show both directions [5-11]. With two exceptions, none of these included structural assessments so any overlap of functional and structural changes could not be determined. One exception [12] reported limited overlap for a mirror reading task; the other [13] reported no overlap for a simple visual-motor task.

We conducted a straightforward test of the site-specific hypothesis. We first examined whether cortical thickness (CT) increased after three months of practicing a visual-spatial game, Tetris. Then, in the same subjects, we determined blood oxygen level dependent (BOLD) changes after practice. Finally, we examined the overlap of the structural and functional results. Since Tetris involves rapid visual-spatial rotation problem-solving and motor co-ordination, we hypothesized GM increases in pre-motor and post-central gyri and parts of the parietal and occipital lobes as shown in previous studies [5,14-16].

## Methods

### Ethics Statement

Each participant gave written assent and a parent gave written informed consent as approved by the University of New Mexico Institutional Review Board. This research was conducted in accord with the Helsinki Declaration.

### Subjects and Procedure

Because developing brains are most likely to show potential changes, we studied adolescents. We chose girls to minimize bias based on previous video game experience.

Twenty-six girls aged 12-15 were recruited from advertisements and visits to schools. All were familiar with computers but none reported significant interest in computer game play (defined as playing any games requiring rapid visual-spatial puzzle solving on a regular basis) or significant exposure to Tetris (defined as playing more than one time, or watching someone else play more than three times). None had medical illness, brain injury, psychiatric history or were taking prescription medications. Girls were randomly assigned to either the Tetris practice group (n = 15) or the control group (n = 11). The groups did not differ on age (mean 13.1 +/- 1.1 vs. 12.9 +/- 1.0, respectively, t = .51, NS), or FSIQ (113.8 +/- 13.4 vs. 115.7 +/- 11.4, t = .40, NS).

The Tetris group got online access to a controlled web site where they logged in to play. Each login was tracked for duration and scores. Each girl received Tetris instruction and 15 minutes of practice when they entered the study. They were instructed to practice as their schedules permitted during a three-month period. On average, the girls practiced 1.5 hours per week (individual games take only a few minutes). The Control group had no access to the Tetris site and was instructed not to play Tetris during the three-month period. Both groups had a structural MRI at the start and end of the study. Both groups also had fMRI scans, while playing Tetris, at both MRI sessions. Before beginning fMRI, both groups received Tetris instructions and practiced 15 minutes.

### Tetris Task

In random order, pieces with different shapes constructed from 4 squares, appear one at a time at the top of the computer screen. As the piece falls to the bottom, the player uses four buttons to move and/or rotate the piece so it fits in with previous pieces to form rows with no gaps. When a row is completed, it disappears from the screen, and all the remaining pieces drop down, changing the configuration of the play space. This requires rapid analysis of the best way to place each piece into the existing pattern. The version used here was scored as time it took to complete 40 rows.

### MRI Acquisition

Images were obtained on a 3 T Siemens TrioTim scanner. Structural (sMRI) images were collected using a 5 echo mprage sequence [TE1 = 1.64 ms, TE2 = 3.5 ms, TE3 = 5.36 ms, TE4 = 7.22 ms, TE5 = 9.08 ms; TR = 2530 ms; FOV = 256 mm; total acquisition time = 6:03]. 192 3D 1 mm thick slices (0 mm skip) were selected to provide coverage of the entire brain (voxel size: 1 mm × 1 mm × 1 mm). Functional echo planar (fMRI) images were collected using a single-shot sequence [TE = 29 ms; TR = 2000 ms; FOV = 24 cm; matrix size = 64 × 64; 32 axial 3.5 mm slices (1.05 mm skip); voxel size: 3.75 × 3.75 × 4.55].

### MR Image Processing

#### Cortical thickness

Structural images were submitted to the CIVET pipeline (1.1.9)  for fully automated analysis. Steps, detailed elsewhere [17], include: 1) Registering to MNI space. 2) Producing high-resolution hemispheric surfaces with 40962 vertices each. 3) Registering surfaces to a high-resolution template. 4) Applying a reverse of step 1 allowing CT estimations in native space of each subject. 5) Smoothing using 20-milimeter kernel [18]. 6) Calculating CT at each vertex.

#### Brain function

Subjects alternated between playing Tetris (ON) for 30 seconds using an MRI compatible device and keeping their eyes fixed (OFF) on a (+) sign for 20 seconds. Each run consisted of 11 ON/OFF cycles totalling 9 minutes 10 seconds per run. On both visits, subjects performed two runs, averaged together for analyses. fMRI images were motion corrected, and normalized to the MNI stereotaxic space using SPM  and re-sampled to 4 × 4 × 4 mm. Data for which motion parameters deviated >3 S.D. from the mean were excluded leaving 11 Tetris and 9 control subjects. Deconvolution determined the hemodynamic response function (HRF) corresponding to the 50-second ON/OFF period and was converted to percent signal change versus baseline. Empirical examination of this 50 second HRF in responsive regions showed large percent signal changes from 4 to 36 seconds into the ON/OFF period. The mean percent signal change from 4-36 seconds, encompassing the theoretical peak of the hemodynamic response [19], was used as a single measure of percent signal change per voxel.

### Statistical Analyses

#### sMRI

**Figure 1:**
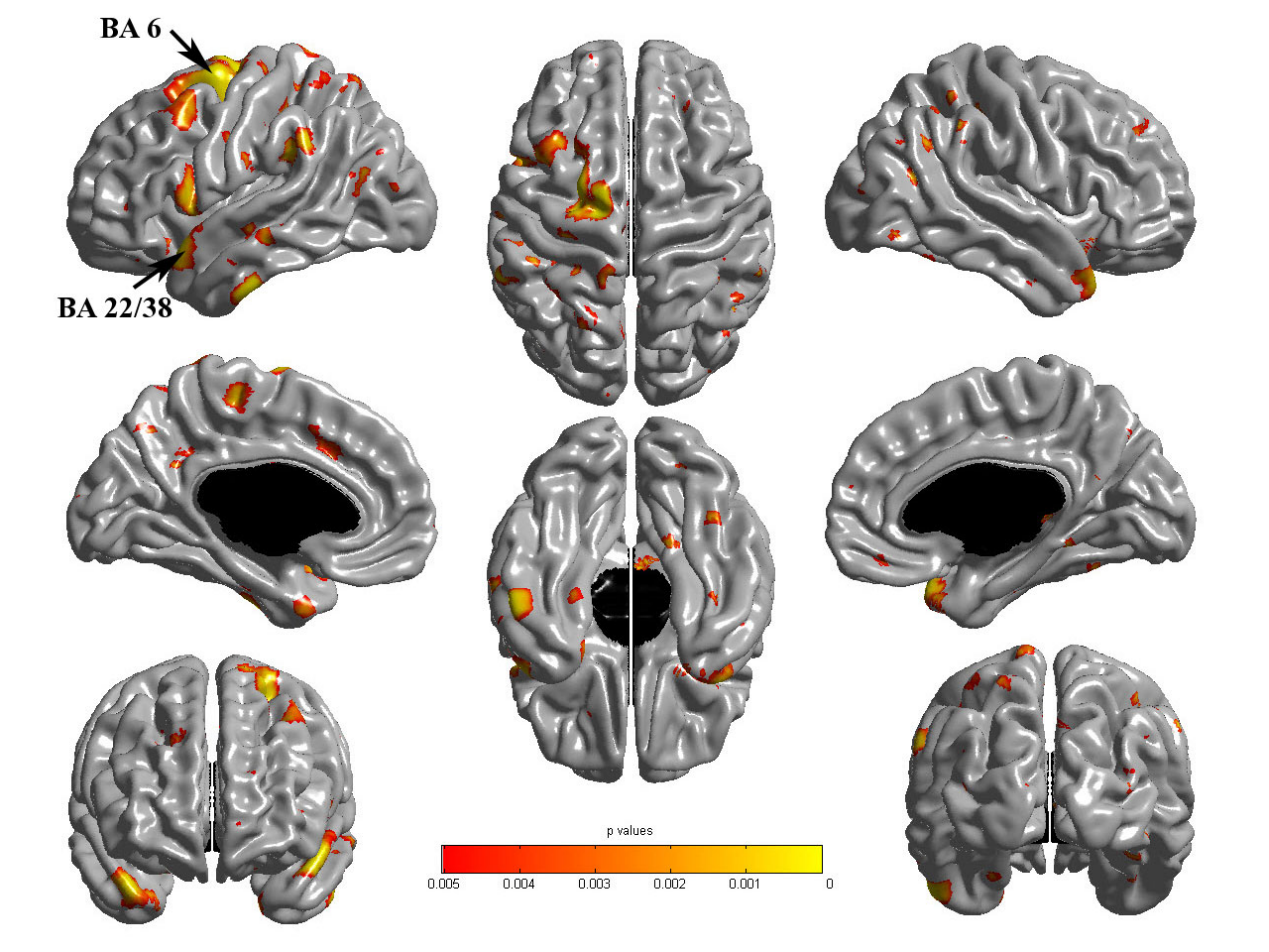
**Cortical Thickness Changes**. Cortical thickness changes after practice showing Tetris follow-up minus baseline versus Controls follow-up minus baseline, p < .005. Upper left is left hemisphere; arrows show cluster level p < .05 FWE corrected.

Analyses used SurfStat created for MATLAB 7 (The MathWorks, Inc.). Baseline cortical thickness maps were subtracted, for each subject, from the 3-month cortical thickness maps, yielding subject-specific maps of change in cortical thickness. These maps for the Tetris group were compared to those for the Control group. Results are shown at p < .05 using the Family Wise Error (FWE) correction (Figure 1); trends are noted at p < .005, uncorrected. For all tables, Brodmann areas (BA) were determined as best estimates based on the Talairach and Tourneau atlas [20,21].

#### fMRI

**Figure 2:**
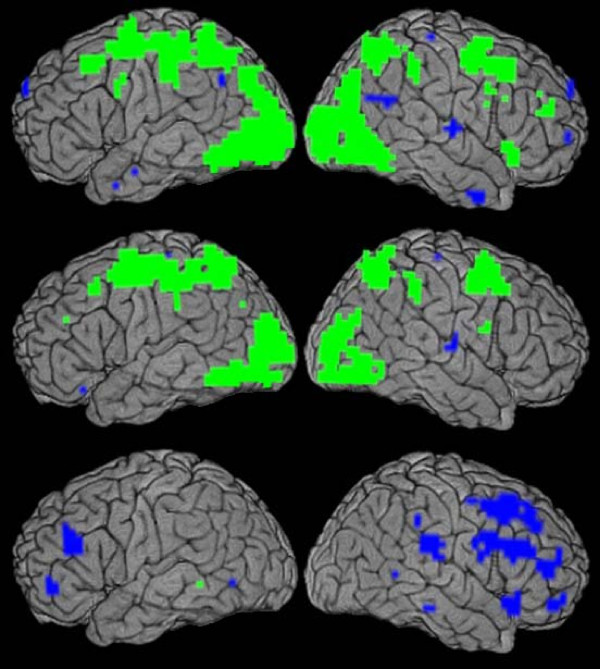
**BOLD Signal Changes**. BOLD signal increases (green) and decreases (blue) in cortex during the Tetris task (top row is at baseline; middle row is at follow-up; p < .05, FWE) and after practice (follow-up minus baseline; bottom row; p < .05, FDR). Left column is left hemisphere. Template is ICBM-152.

Individual subject voxel-wise percent BOLD signal change in response to Tetris was passed on to level 2 group analysis. One sample t-tests (df = 10 for Tetris group, df = 8 for controls, SPM) against the null hypothesis showed areas significantly modulated by Tetris (p < 0.05; Figure 2, top two panels). Practice effects in the Tetris group were assessed using a paired two-sample t-test contrasting the follow-up vs. baseline visits (Figure 2, lower panel). Group-by-visit interactions were assessed using an unpaired t-test (df = 18) contrasting (Percent signal change at 3 months - Baseline Percent signal change in Experimental Group) Minus (Percent signal change at 3 months - Baseline Percent signal change in Experimental Group). Stereotaxic coordinates of peak statistical significance were converted from ICBM-152 to Talairach compatible coordinates [21].

We limit discussion to the more conservative FWE results since the chance of Type I error is greater for results based on more lenient threshold methods of FDR or uncorrected p values. Nonetheless, we included these findings in the tables to increase the chance of replication by other studies.

## Results

Changes in CT following practice led to a significantly thicker cortex in the experimental compared to the control group [Tetris follow-up minus baseline] minus [control follow-up minus baseline], in two Brodmann areas (BA), left BA 6 and left BA 22/38, (p < .05, FWE corrected); these are shown in Figure 1 along with other areas where there was a trend of increases after practice (p < .005, uncorrected). These areas were distributed throughout the brain (Table 1) and were most significant (p < .0005, uncorrected) in BA 6 and 44 (left frontal lobe), BA 40 (bilateral parietal lobe), BA 4 (left paracentral lobule), BA 20 and 22 (left temporal lobe), and BA 38 (right temporal pole). There was no evidence of any areas of thinner cortex after practice.

**Table 1:** Peak vertex coordinates (MNI) and brain areas where change over three months showed greater cortical thickness in the Tetris group than in the controls (p < 0.005, uncorrected; * p < .05 cluster level, FWE corrected).

**Brodmann Area**	**Region Name**	**X, Y, Z Coordinates**	**P Value**
**Left Frontal**			
BA 6	Superior frontal gyrus	-13, -10, 69	0.00004
BA 6	Superior frontal gyrus	-24, 0, 67	0.00007*
BA 6	Superior frontal gyrus	-15, -4, 74	0.0001
BA 6, 8	Middle frontal gyrus	-44, 14, 45	0.0013
BA 6, 44	Inferior frontal gyrus	-52, 12, 10	0.0005
BA 45, 46	Inferior frontal gyrus	-41, 41, 5	0.004
**Right Frontal**			
BA 9	Superior frontal gyrus	20, 39, 39	0.002
**Left Parietal**			
BA 7	Superior parietal lobule	-36, -51, 64	0.003
BA 7	Superior parietal lobule	-24, -67, 63	0.002
BA 7	Superior parietal lobule	-13, -45, 76	0.002
BA 5	Superior parietal lobule	-25, -41, 60	0.002
BA 19, 39	TemporoParietoOccipital junction	-44, -71, 19	0.002
BA 40	Supramarginal gyrus	-63, -42, 46	0.0004
BA 31	Precuneus	-5, -64, 45	0.002
BA 31	Precuneus	-10, -53, 29	0.003
BA 4	Paracentral Lobule	-7, -25, 61	0.0001
**Right Parietal**			
BA 40	Supramarginal gyrus	61, -41, 43	0.0002
BA 39	Angular gyrus	47, -57, 34	0.001
BA 39	Angular gyrus	50, -52, 49	0.004
BA 7	Superior parietal lobule	41, -47, 55	0.0009
**Left Temporal**			
BA 22/38	Anterior superior temporal gyrus	-50, 16, -16	0.00008*
BA 20	Anterior Inferior temporal gyrus	-58, -15, -35	0.0002
BA 21	Middle temporal gyrus	-65, -25, -12	0.001
BA 38	Medial temporal pole	-22, 7, -38	0.002
BA 28	Parahippocampal gyrus	-28, -20, -29	0.002
**Right Temporal**			
BA 38	Temporal pole	41, 22, -29	0.0004
BA 37	Posterior inferior temporal gyrus	39, -58, -20	0.002
BA 37	Posterior inferior temporal gyrus	45, -72, -9	0.0008
BA 36	Lingual gyrus	19, -43, -10	0.001
**Left Occipital**			
BA 19	Lateral occipital gyrus	-40, -85, 11	0.003
**Right Occipital**			
BA 19	Lateral occipital gyrus	37, -88, 14	0.002
**Left Cingulate**			
BA 24	Anterior cingulate gyrus	-9, 19, 38	0.004
BA 23, 31	Posterior cingulate gyrus	-4, -38, 27	0.001

Tetris-induced BOLD signal increases (p < 0.05, FWE corrected) are similar for both visits (Figure 2, top two panels; Additional Files 1, 2, 3 &4). Most are in frontal and parietal lobes including parts of BAs 6, 7, 40, although the follow-up visit showed additional activations in occipital BAs 17, 18, and 19. For both visits, only a few small clusters (<20 voxels each) showed a significantly decreased BOLD signal during task performance. The same comparisons for the control group showed similar results.

For the Tetris group, no activation changes over the 3-month practice period were significant after FWE correction. However, changes based on statistical false discovery rate control (FDR) [22,23] are shown in Figure 2 (lower panel) and Table 2. These were all decreases after practice and were mostly in the right hemisphere; the most significant were in frontal BAs 32, 8, 9, 6, 46, and in parietal BA 40.

**Table 2:** Brain areas with decreases in BOLD signal after practice (follow-up minus baseline; p < .05 FDR).

**Brodmann Area**	**Region Name**	**X, Y, Z Coordinates (MNI)**	**P Value**
**Left Frontal**			
BA 9	Middle Frontal Gyrus	-38, 30, 26	0.031
BA 46	Inferior Frontal Gyrus	-46, 42, -2	0.038
**Right Frontal**			
BA 32	Medial Frontal Gyrus	10, 18, 46	0.026
BA 8	Superior Frontal Gyrus	6, 34, 46	0.026
BA 6	Middle Frontal Gyrus	42, 6, 50	0.031
BA 46	Middle Frontal Gyrus	50, 42, 14	0.032
BA 9	Middle Frontal Gyrus	50, 26, 26	0.033
BA 6	Precentral Gyrus	58, 6, 34	0.035
BA 47	Inferior Frontal Gyrus	38, 22, -14	0.033
*	Sub-Gyral	38, 50, -10	0.033
BA 6	Medial Frontal Gyrus	6, -6, 54	0.039
BA 6	Precentral Gyrus	38, 2, 30	0.046
**Right Parietal**			
BA 40	Inferior Parietal Lobule	66, -30, 26	0.026
BA 40	Sub-Gyral	38, -38, 38	0.043
BA 40	Inferior Parietal Lobule	58, -38, 38	0.049
BA 40	Postcentral Gyrus	58, -22, 18	0.05
**Left Occipital**			
BA 19	Middle Occipital Gyrus	-58, -66, -2	0.038
**Right Temporal**			
BA 22	Superior Temporal Gyrus	50, -26, -14	0.044
BA 22	Superior Temporal Gyrus	58, -50, 6	0.049
**Right Cingulate**			
BA 24	Cingulate Gyrus	10, 14, 30	0.038
BA 24	Anterior Cingulate	10, 30, 22	0.038

**Figure 3:**
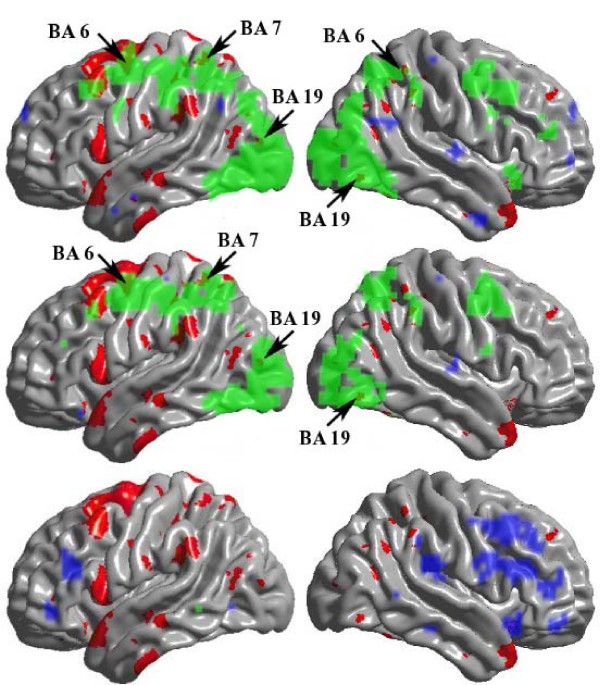
**Cortical Thickness and BOLD Changes**. Both cortical thickness change after practice (red) and BOLD signal increases (green) or decreases (blue) during Tetris task are shown. Top row is at baseline, middle row is at follow-up, bottom row is follow-up minus baseline. Left column is left hemisphere. Template is Surfstat MNI. Arrows indicate small areas of overlap.

Figure 3 shows both CT change and BOLD signal during Tetris play. For baseline and follow-up, overlap was limited to a few small areas of BAs 6, 7, 22, and 19 (arrows in Figure 3, top and middle rows). The areas with decreased activation over the practice period (follow-up minus baseline) showed no overlap with CT increases (bottom row). To increase statistical power, we did additional analyses, first restricting the CT analyses to regions that showed BOLD changes; and second, restricting the BOLD analyses to regions that showed CT changes. Neither analysis identified any new regions of overlap (p < .005, uncorrected).

## Discussion

We found no apparent overlap between structural and functional findings to support the site-specific hypothesis. Interpreting fMRI data has important limitations. For example, the BOLD signal reflects neuronal mass activity. It does not distinguish between excitatory and inhibitory activity, and a marked change in processing strategies involving an anatomical area does not necessitate a change in BOLD [24]. It is possible that practiced-subjects developed a more sophisticated approach, but if this did not invoke novel brain areas, it might not be detectable using fMRI.

The frontal eye fields in left BA 6 showed the most significant CT change, consistent with other imaging studies of visual-spatial tasks. The same area also showed increased activity while the subjects played at baseline, but there was no additional change after practice. Notably, BA 6 is at a critical juncture between "intention" and "action" [25], linking memory of previous responses and the rewards associated with them [26], and is active when motor plans are altered [27]. The left temporal pole (BA 22/38) also showed change. The temporal poles integrate visual, auditory, tactile, and internal physiological information, suggesting a critical role in multimodal perceptual analysis [28]. Moreover, there are lateralizing differences, with right more associated with emotion and socially relevant episodic memories, and left associated with semantic memory[29]. We interpret the temporal pole changes as reflecting increased multisensory integration to support visual, spatial, and tactile input necessary to succeed at Tetris. That greater cortical change was seen in the left temporal pole, suggests Tetris play is processed as a general cognitive puzzle rather than a discrete episodic memory. Whether practiced Tetris play and associated cortical changes generalize to performance changes in other cognitive domains (e.g. working memory, processing speed, spatial reasoning) is not yet determined.

## Conclusion

These data indicate that structural and functional brain changes after practice do not necessarily occur in the same location, at least on the levels assessed with these MRI methods.

## Competing interests

This project was funded at the MIND Research Network, Albuquerque, N.M. by Blue Planet Software (BPS), Inc., the company holding exclusive licensing rights to Tetris. REJ was the PI and LL received partial salary support. The funders had no role in study design, data collection and analysis, decision to publish, or preparation of the manuscript. RJH is a paid consultant to Blue Planet Software.

## Authors' contributions

RJH and REJ conceived and designed the experiment. REJ performed the experiment. SK and LL analyzed the data. All authors approved and helped write the manuscript.

## Supplementary Material

Additional file 1 **Brain areas of increased BOLD signal during task in the Tetris group at baseline (p < .05 FWE)**. Functional activations while playing Tetris before practice period. Click here for file

Additional file 2 **Brain areas of decreased BOLD signal during task in the Tetris group at baseline (p < .05 FWE)**. Functional deactivations while playing Tetris before practice period. Click here for file

Additional file 3 **Brain areas of increased BOLD signal during task in the Tetris group at follow-up (p < .05 FWE)**. Functional activations while playing Tetris after practice period. Click here for file

Additional file 4 **Brain areas of decreased BOLD signal during task in the Tetris group at follow-up (p < .05 FWE)**. Functional deactivations while playing Tetris after practice period. Click here for file
