# Supplementary material for: MRI assessment of cortical thickness and functional activity changes in adolescent girls following three months of practice on a visual-spatial task
Source: BMC Res Notes. 2009 Sep 1;2:174. doi: 10.1186/1756-0500-2-174 (PMC2746806; doi:10.1186/1756-0500-2-174)
Supplement: Additional file 1 — Brain areas of increased BOLD signal during task in the Tetris group at baseline (p < .05 FWE). Functional activations while playing Tetris before practice period. [file 1756-0500-2-174-S1.doc]

# Additional File 1. Brain areas of increased BOLD signal during task in the Tetris group at baseline (p<.05 FWE).

| **Brodmann Area** | **Region Name** | **X, Y, Z Co-ordinates (MNI)** | **P Value** |
| --- | --- | --- | --- |
| **Left Frontal** |  |  |  |
| BA 6 | Middle Frontal Gyrus | -22, -16, 62 | 0 |
| BA 6 | Sub-Gyral | -22, 2, 58 | 0 |
| BA 6 | Medial Frontal Gyrus | 2, 18, 46 | 0 |
| BA 6 | Precentral Gyrus | -50, 2, 34 | 0.007 |
| BA 4 | Precentral Gyrus | -50, -2, 42 | 0.007 |
| **Right Frontal** |  |  |  |
| BA 9 | Middle Frontal Gyrus | 38, 46, 22 | 0 |
| BA 6 | Middle Frontal Gyrus | 30, -2, 54 | 0 |
| BA 6 | Superior Frontal Gyrus | 30, 2, 66 | 0.003 |
| BA 6 | Superior Frontal Gyrus | 18, -2, 66 | 0.016 |
| BA 6 | Middle Frontal Gyrus | 50, 6, 38 | 0.014 |
| BA 6 | Precentral Gyrus | 50, 6, 26 | 0.042 |
| BA 8 | Middle Frontal Gyrus | 38, 38, 34 | 0.045 |
| **Left Parietal** |  |  |  |
| BA 40 | Inferior Parietal Lobule | -50, -30, 54 | 0 |
| BA 7 | Precuneus | -22, -54, 58 | 0 |
| BA 7 | Precuneus | -18, -66, 50 | 0 |
| **Right Parietal** |  |  |  |
| BA 7 | Precuneus | 18, -66, 54 | 0 |
| BA 7 | Precuneus | 14, -58, 58 | 0 |
| BA 7 | Precuneus | 22, -54, 54 | 0 |
| BA 7 | Precuneus | 34, -42, 54 | 0.007 |
|  | Sub-Gyral | 34, -38, 42 | 0.009 |
| **Left Temporal** |  |  |  |
| BA 27 | Parahippocampal Gyrus | 22, -30, -6 | 0.037 |
| **Left Occipital** |  |  |  |
| BA 18 | Middle Occipital Gyrus | -42, -82, -6 | 0 |
| **Right Occipital** |  |  |  |
| BA 19 | Middle Occipital Gyrus | 42, -74, 6 | 0 |
| **Right Cingulate** |  |  |  |
| BA 24 | Cingulate Gyrus | 10, 18, 30 | 0.03 |
| **Left Subcortical** |  |  |  |
| Ventral Posterior Medial Nucleus | Thalamus | -14, -18, 6 | 0.004 |
| **Right Subcortical** |  |  |  |
| BA 13 | Insula | 34, 26, -6 | 0.001 |
| Lateral Dorsal Nucleus | Thalamus | 14, -18, 18 | 0.016 |
