# Supplementary material for: MRI assessment of cortical thickness and functional activity changes in adolescent girls following three months of practice on a visual-spatial task
Source: BMC Res Notes. 2009 Sep 1;2:174. doi: 10.1186/1756-0500-2-174 (PMC2746806; doi:10.1186/1756-0500-2-174)
Supplement: Additional file 2 — Brain areas of decreased BOLD signal during task in the Tetris group at baseline (p < .05 FWE). Functional deactivations while playing Tetris before practice period. [file 1756-0500-2-174-S2.doc]

Additional File 2. Brain areas of decreased BOLD signal during task in the Tetris group at baseline (p<.05 FWE).

| **Brodmann Area** | **Region Name** | **X, Y, Z Co-ordinates (MNI)** | **P Value** |
| --- | --- | --- | --- |
| **Left Frontal** |  |  |  |
| BA 8 | Medial Frontal Gyrus | -2, 58, 38 | 0.001 |
| **Right Frontal** |  |  |  |
| BA 10 | Medial Frontal Gyrus | 10, 58, 6 | 0.007 |
| **Right Parietal** |  |  |  |
| BA 3 | Post Central Gyrus | 22, -30, 66 | 0.035 |
| **Left Temporal** |  |  |  |
| BA 39 | Middle Temporal Gyrus | -50, -62, 34 | 0.006 |
| BA 21 | Inferior Temporal Gyrus | -62, -6, -18 | 0.025 |
| BA 21 | Middle Temporal Gyrus | -58, 6, -26 | 0.045 |
| **Right Temporal** |  |  |  |
| BA 21 | Middle Temporal Gyrus | 58, 2, -34 | 0.004 |
| **Left Occipital** |  |  |  |
| BA 31 | Precuneus | -6, -66, 26 | 0.023 |
| **Left Cingulate** |  |  |  |
| BA 31 | Posterior Cingulate | -6, -50, 26 | 0.008 |
| **Right Insula** |  |  |  |
| BA 13 | Insula | 42, -14, 10 | 0.001 |
