# Supplementary material for: MRI assessment of cortical thickness and functional activity changes in adolescent girls following three months of practice on a visual-spatial task
Source: BMC Res Notes. 2009 Sep 1;2:174. doi: 10.1186/1756-0500-2-174 (PMC2746806; doi:10.1186/1756-0500-2-174)
Supplement: Additional file 3 — Brain areas of increased BOLD signal during task in the Tetris group at follow-up (p < .05 FWE). Functional activations while playing Tetris after practice period. [file 1756-0500-2-174-S3.doc]

Additional File 3. Brain areas of increased BOLD signal during task in the Tetris group at follow-up (p<.05 FWE).

| **Brodmann Area** | **Region Name** | **X, Y, Z Co-ordinates (MNI)** | **P Value** |
| --- | --- | --- | --- |
| **Left Frontal** |  |  |  |
| BA 6 | Sub-Gyral | -22, 2, 54 | 0 |
| BA 32 | Medial Frontal Gyrus | 2, 14, 46 | 0.002 |
| BA 9 | Middle Frontal Gyrus | -38, 34, 30 | 0.04 |
| **Right Frontal** |  |  |  |
| BA 6 | Sub-Gyral | 26, 2, 54 | 0 |
| BA 6 | Precentral Gyrus | 50, 6, 26 | 0.012 |
| **Left Parietal** |  |  |  |
| BA 40 | Postcentral Gyrus | -42, -26, 54 | 0 |
| BA 7 | Superior Parietal Lobule | -22, -58, 62 | 0.001 |
| **Right Parietal** |  |  |  |
| BA 7 | Precuneus | 18, -70, 58 | 0 |
| BA 7 | Precuneus | 22, -66, 50 | 0.001 |
| BA 7 | Precuneus | 22, -58, 54 | 0.002 |
| BA 7 | Precuneus | 34, -42, 54 | 0.005 |
|  | Sub-Gyral | 34, -38, 42 | 0.004 |
| **Left Occipital** |  |  |  |
| BA 18 | Middle Occipital Gyrus | -30, -94, 10 | 0 |
| BA 18 | Middle Occipital Gyrus | -34, -94, 18 | 0 |
| BA 18 | Inferior Occipital Gyrus | -38, -86, -10 | 0 |
| BA 17 | Lingual Gyrus | -10, -98, 2 | 0.003 |
| BA 31 | Precuneus | -26, -74, 34 | 0.044 |
| **Right Occipital** |  |  |  |
| BA 19 | Middle Occipital Gyrus | 38, -86, 10 | 0 |
| BA 17 | Lingual Gyrus | 26, -90, 2 | 0 |
| BA 17 | Lingual Gyrus | 14, -90, 2 | 0 |
| **Left Subcortical** |  |  |  |
| Substania Nigra | * | -14, -22, -6 | 0 |
| Red Nucleus | * | -6, -26, -6 | 0 |
| Lateral Globus Pallidus | Lentiform Nucleus | -14, -2, 2 | 0.004 |
| **Right Subcortical** |  |  |  |
| * | Thalamus | 22, -30, -2 | 0 |
| * | Thalamus | 10, -14, 18 | 0.031 |
