# Supplementary material for: MRI assessment of cortical thickness and functional activity changes in adolescent girls following three months of practice on a visual-spatial task
Source: BMC Res Notes. 2009 Sep 1;2:174. doi: 10.1186/1756-0500-2-174 (PMC2746806; doi:10.1186/1756-0500-2-174)
Supplement: Additional file 4 — Brain areas of decreased BOLD signal during task in the Tetris group at follow-up (p < .05 FWE). Functional deactivations while playing Tetris after practice period. [file 1756-0500-2-174-S4.doc]

Additional File 4. Brain areas of decreased BOLD signal during task in the Tetris group at follow-up (p<.05 FWE)

| **Brodmann Area** | **Region Name** | **X, Y, Z Co-ordinates (MNI)** | **P Value** |
| --- | --- | --- | --- |
| **Left Frontal** |  |  |  |
| BA 47 | Inferior Frontal Gyrus | -34, 26, -14 | 0.002 |
| **Right Frontal** |  |  |  |
| BA 4 | Precentral Gyrus | 18, -26, 66 | 0.033 |
| **Left Parietal** |  |  |  |
| BA 4 | Postcentral Gyrus | -14, -26, 66 | 0.012 |
| **Right Insula** |  |  |  |
| BA 13 | Insula | 46, -14, 10 | 0.008 |
